# Optimal definition of biological tumor volume using positron emission tomography in an animal model

**DOI:** 10.1186/s13550-015-0134-y

**Published:** 2015-10-21

**Authors:** Ingrid Wu, Hao Wang, David Huso, Richard L. Wahl

**Affiliations:** Department of Radiology, Division of Nuclear Medicine, The Johns Hopkins University School of Medicine, Baltimore, MD USA; Division of Oncology Biostatistics and Bioinformatics, The Johns Hopkins University School of Medicine, Baltimore, MD USA; Department of Molecular and Comparative Pathobiology, The Johns Hopkins University School of Medicine, Baltimore, MD USA; Department of Radiology, Washington University School of Medicine, Campus Box 8131, 660S, Euclid Ave, St. Louis, MO 63110 USA

## Abstract

**Background:**

The goal of the study is to investigate ^18^F-fluorodeoxyglucose positron emission tomography (^18^F-FDG-PET)’s ability to delineate the viable portion of a tumor in an animal model using cross-sectional histology as the validation standard.

**Methods:**

Syngeneic mammary tumors were grown in female Lewis rats. Macroscopic histological images of the transverse tumor sections were paired with their corresponding FDG micro-PET slices of the same cranial-caudal location to form 51 pairs of co-registered images. A binary classification system based on four FDG-PET tumor contouring methods was applied to each pair of images: threshold based on (1) percentage of maximum tumor voxel counts (C_max_), (2) percentage of tumor peak voxel counts (C_peak_), (3) multiples of liver mean voxel counts (C_liver_) derived from PERCIST, and (4) an edge-detection-based automated contouring system. The sensitivity, which represented the percentage of viable tumor areas correctly delineated by the gross tumor area (GTA) generated from a particular tumor contouring method, and the ratio (expressed in percentage) of the overestimated areas of a gross tumor area (GTA_OE_)/whole tumor areas on the macroscopic histology (WTA_H_), which represented how much a particular GTA extended into the normal structures surrounding the primary tumor target, were calculated.

**Results:**

The receiver operating characteristic curves of all pairs of FDG-PET images have a mean area under the curve value of 0.934 (CI of 0.911–0.954), for representing how well each contouring method accurately delineated the viable tumor area. FDG-PET single value threshold tumor contouring based on 30 and 35 % of tumor C_max_ or C_peak_ and 6 × C_liver_ + 2 × SD achieved a sensitivity greater than 90 % with a GTA_OE_/WTA_H_ ratio less than 10 %. Contouring based on 50 % of C_max_ or C_peak_ had a much lower sensitivity of 67.2–75.6 % with a GTA_OE_/WTA_H_ ratio of 1.1–1.7 %. Automated edge detection was not reliable in this system.

**Conclusions:**

Single-value-threshold tumor contouring using ^18^F-FDG-PET is able to accurately delineate the viable portion of a tumor. 30 and 35 % of C_max_, 30 and 35 % of C_peak_, and 6 × C_liver_ + 2 × SD are three appropriate threshold values to delineate viable tumor volume in our animal model. The commonly used threshold value of 50 % of C_max_ or C_peak_ failed to detect one third of the viable tumor volume in our model.

## Background

^18^F-FDG-PET can identify local and disseminated malignant lesions and help separate tumor from surrounding normal tissue. Accurate tumor delineation based on FDG-PET imaging is crucial for PET based radiotherapy planning. A key issue is to determine how to best delineate the tumor/non-tumor margin from FDG-PET images. In general, the contouring methods used in these studies can be grouped into several approaches: “top-down,” “bottom-up,” “source-to-background ratio,” or “edge detection” [1–6].

In a top-down approach, a fixed percentage of the highest tumor standard uptake value (SUV) is applied to the FDG-PET image. Fifty percent of the maximum SUV in the tumor was reported as the best threshold for delineation of non-small cell lung cancer (NSCLC) tumors by Wu et al. [2]. Erdi et al. suggested 42 % of the maximum tumor uptake intensity as a proper contouring value in phantom studies [3]. Using a technique similar to Erdi’s, van Baardwijk et al. obtained a significant correlation between FDG-PET volume and histopathological measurements for 23 NSCLC tumors [4].

In a bottom-up approach, tumor delineation is based on normal tissue or the background SUV. Zasadny et al. used a bottom-up approach in lung cancer, where they selected FDG-PET voxel intensities that were over three times the standard deviation above the intensity of the normal lung background as the target tumor volume [5].

Finally, broadly based on edge detection, several institutions have investigated their own computerized algorithms derived from mathematical models. Hatt et al. have developed a fuzzy locally adaptive Bayesian algorithm to compare maximum diameters of delineated volumes on FDG-PET to a histopathological sample [7].

Some studies have left the decision of how to delineate tumor volume in the hands of radiation oncologists and nuclear medicine physicians, using FDG-PET adjunctively to improve the accuracy of tumor volume determined from CT, especially to separate normal tissues from tumor [8].

Another important issue in PET based radiotherapy planning is to determine whether ^18^F-FDG-PET imaging can delineate the viable portion of a heterogeneous tumor from the necrotic portion. The validation standards reported in the literature are too simplified to adequately address this aspect of FDG-PET imaging [6]. In most clinical studies, surgical specimens were used as the “gold standard”, and results derived from the tumor contouring methods were compared to the external dimensions of the surgical specimen. There are two major drawbacks to such a validation standard. First, tumors are usually irregularly shaped, which makes a comparison in dimensions quite coarse. Second, a tumor specimen’s outer dimensions provide no information about the tumor’s internal biological heterogeneity. Tumors of similar dimensions may have different internal distributions of viable tumor tissues, and there are only a few studies taking into account such biological variation [8]. Tumors may shrink during histological processing, which further complicates the overall validation process [9].

The purpose of our study was to design a validation system for FDG-PET tumor contouring method using corresponding cross-sectional pathology based on frozen tumor sections as the gold standard. We also investigated FDG-PET’s ability, using several analytical approaches, to demonstrate a tumor’s internal heterogeneous distribution of viable tumor tissues in addition to delineation of the total tumor volume. We would like to quantify the viable and necrotic regions within a tumor to facilitate animal radiotherapy planning studies seeking dose efficiency and reduction or tumor necrosis targeted radiotherapy [10, 11].

## Methods

This project was approved by the animal care committee. IRB approval was not obtained because this was a pre-clinical study and no human subjects or tissues were involved.

### Tumor model

Carcinogen-induced rat mammary tumor line was selected as the animal model due to their known heterogeneity and syngeneic nature, allowing them to grow in rodents with a competent immune system [12, 13]. Suspension containing approximately 10^6^ cells was sequentially propagated in vivo by implanting them in the scapular fat pad region of female Lewis rats (purchased from Charles River Laboratories). The animal models in our experiment were established from three consecutive generations of propagations in a total of nine Lewis rats.

### Tumor imaging

Rats with mammary tumors were prepared for FDG-PET imaging when the tumors reached at least 3 cm in diameter in about 2–3 weeks. Tumor-bearing rats were shaved in the scapular area and fasted the night before imaging with a Philips MOSAIC Animal PET Imaging System [14]. To maintain identical anatomical orientations during tumor imaging and tumor specimen processing, a stand-alone rat bed was constructed in the same shape as the animal PET scanner imaging bed.

On the day of imaging, tumor-bearing rats were anesthetized with 1 % isoflurane. 3.7 × 10^7^ Bq (1 m Ci) of ^18^F-FDG (P.E.T.Net, Glen Burnie, MD, USA) was injected through a tail vein. After radiotracer injection, the rat was kept warm for a 1-h uptake period until imaging and then placed in the imaging bed in the prone position, sedated, with the tumor on its back positioned upright.

**Fig. 1:**
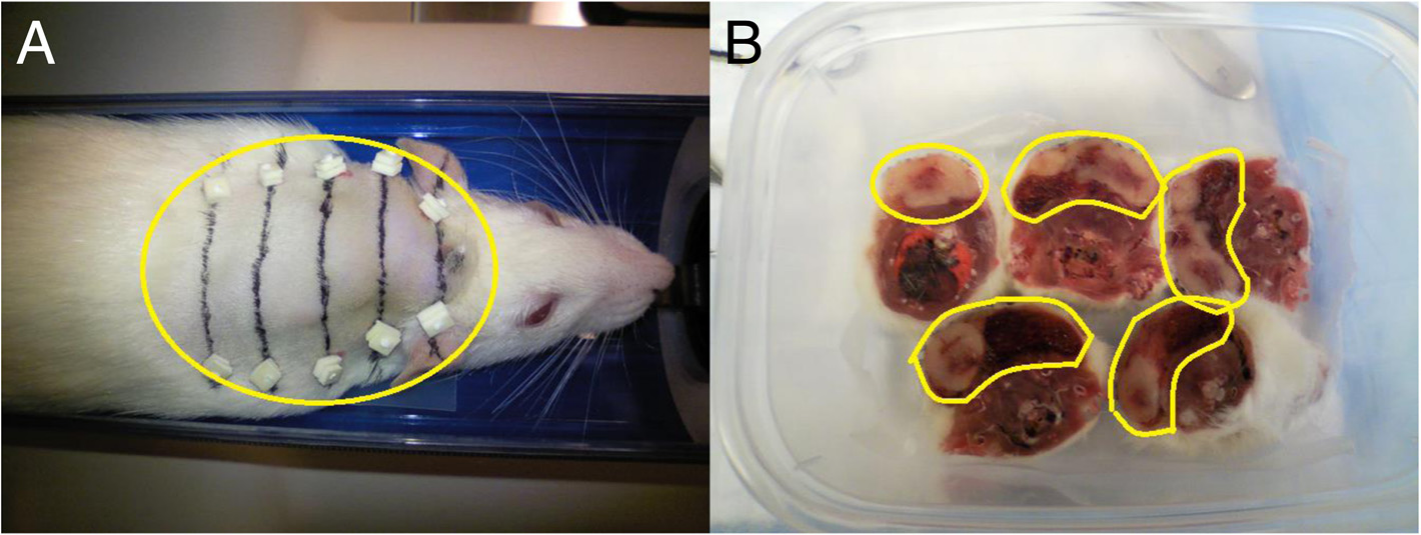
Tumor processing. **a** The rat was anesthetized, transverse markings were drawn across the tumor (*circled in yellow*), and fiducial markers (*white squares*) were placed. **b** After the whole rat was frozen, the portion of the rat containing tumor (with tumor circled in yellow) was sectioned into transverse blocks along the skin markings and placed into an airtight container. The individual sections were then frozen and then analyzed visually for viability with preservation of orientation

Using the PET scanner’s integrated laser positioning system, 3–5 transverse sectioning positions were identified and marked in each rat. At each sectioning position, the skin over the tumor was marked and the imaging bed position recorded. In order to facilitate accurate translational and rotational imaging registration, two fiducial markers with ^18^F-FDG were placed at each transverse sectioning line (Fig. 1a). In order to reconstruct the attenuation corrected FDG-PET images, a transmission scan was performed before the PET scan. The static PET imaging acquisition lasted for 30 min.

The FDG-PET images were reconstructed using three-dimensional row-action maximum-likelihood algorithm on a 128 × 128 × 120 matrix, where the voxel size equals 1 mm^3^. Attenuation was corrected using a ^137^Cs single photon transmission measurement. Decay correction, normalization, dead time correction, and scatter correction were also utilized [12].

### Tumor processing

After imaging, the rat was humanely sacrificed in the imaging bed by gas anesthesia overdosing, with its imaging position maintained. It was then carefully transferred to the identical rat bed with its anatomical orientation preserved. The whole rat and its rat bed were quickly frozen using dry ice and transferred to a −20 °C freezer. In the following 2–3 h, the whole rat was allowed to freeze to the extent where the necrotic portion of the tumor was solidified but could still be sliced into transverse sections with relative ease.

We had a device specially designed to allow easy and accurate sectioning of the rat along its skin markings, so that the sectioning planes parallel to the axial planes of the FDG-PET images. The tumor-containing portion of each rat was sectioned transversely into three to five blocks, and each sectioning resulted in a rostral and a caudal cross-sectional surfaces. In order to preserve the anatomical configuration, the blocks were sectioned at a sufficient thickness (6–8 mm) to prevent block deformity from the shearing force of sectioning. All blocks were stored in an airtight box to avoid moisture and kept in the freezer overnight (Fig. 1b). All cross-sectional surfaces were imaged at a visible light wavelength and digitized using a flat panel color scanner with a resolution of 200 dots per inch (DPI). These images are the macroscopic histological images of tumor blocks.

Rats 1–8 were processed in the method described above, and a total of 51 technically adequate macroscopic histological images were acquired from eight rats. Rat 9, serving as a reference rat, was processed differently. After it was sacrificed on the imaging bed, we harvested its tumor, submerged the tumor specimen in 10 % formalin, and sent it for hematoxylin and eosin (H&E) staining and microscopic histology.

### Image data processing

**Fig. 2:**
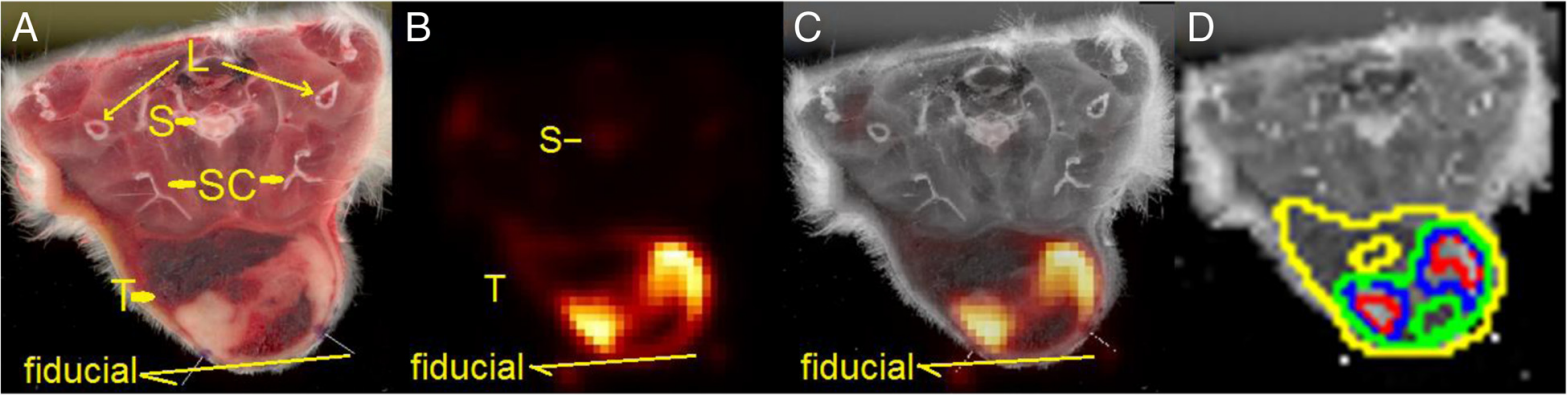
Fused images. Co-registered images from Rat 5 in supine position, with tumor on its back. **a** Transverse section of the rat through limb (L), spine (S), scapular (SC), and tumor (T). The *central white portion* of the tumor represents viable tumor areas, while the more *peripheral red portion* of the tumor represents necrotic tumor areas with hemorrhage. **b** The corresponding FDG-PET image. **c** The fused images of **a** and **b**, where the FDG-PET image voxel signal intensities closely correlate with the geographic distribution of the viable and necrotic tumor areas. Note the well-overlapped fiducial markers on the dorsal aspect of the tumor. **d** Four gross tumor areas (GTAs) generated from variable percentages of tumor peak voxels (C_peak_) as well as PERCIST (1.5 × C_liver mean_ + 2 × Cliver SD) were overlaid onto the histological image: *Red* represented 70 % of C_peak_, *blue* represented 50 % of C_peak_, *green* represented 30 % of C_*max*_, and *yellow* represented the PERCIST based threshold. Among the four GTAs, 30 % C_peak_ (*green*) best delineated the viable portion of the tumor

There were two categories of image data to be processed: (1) Macroscopic histological images generated from cross-sectional surfaces of the frozen tumor blocks, as demonstrated in Fig. 1b and Fig. 2a. (2) The reconstructed FDG-PET imaging slices of the same tumor at the identical cranial-caudal level as the histological images from the first category of data, as demonstrated in Fig. 2b.

A note needed to be made on the comparison between the macroscopic histological image and the FDG-PET image. Each FDG-PET slice represented a three-dimensional (3D) distribution of radioactivity. Ideally, the corresponding histological gold standard should have been a very thin piece of 3D tissue block of the same thickness, and the comparison should have been made in volume instead of area. However, to produce a tissue block of 1-mm thickness and measure its viable and necrotic volumes is technically difficult. Instead, we used the macroscopic histological image of the tumor’s cross-sectional surface—a two-dimentional (2D) image (as demonstrated in Fig. 2a) with the assumption that there was modest macroscopic variation in the distribution of viable tumor tissues within a 1-mm thickness.

**Fig. 3:**
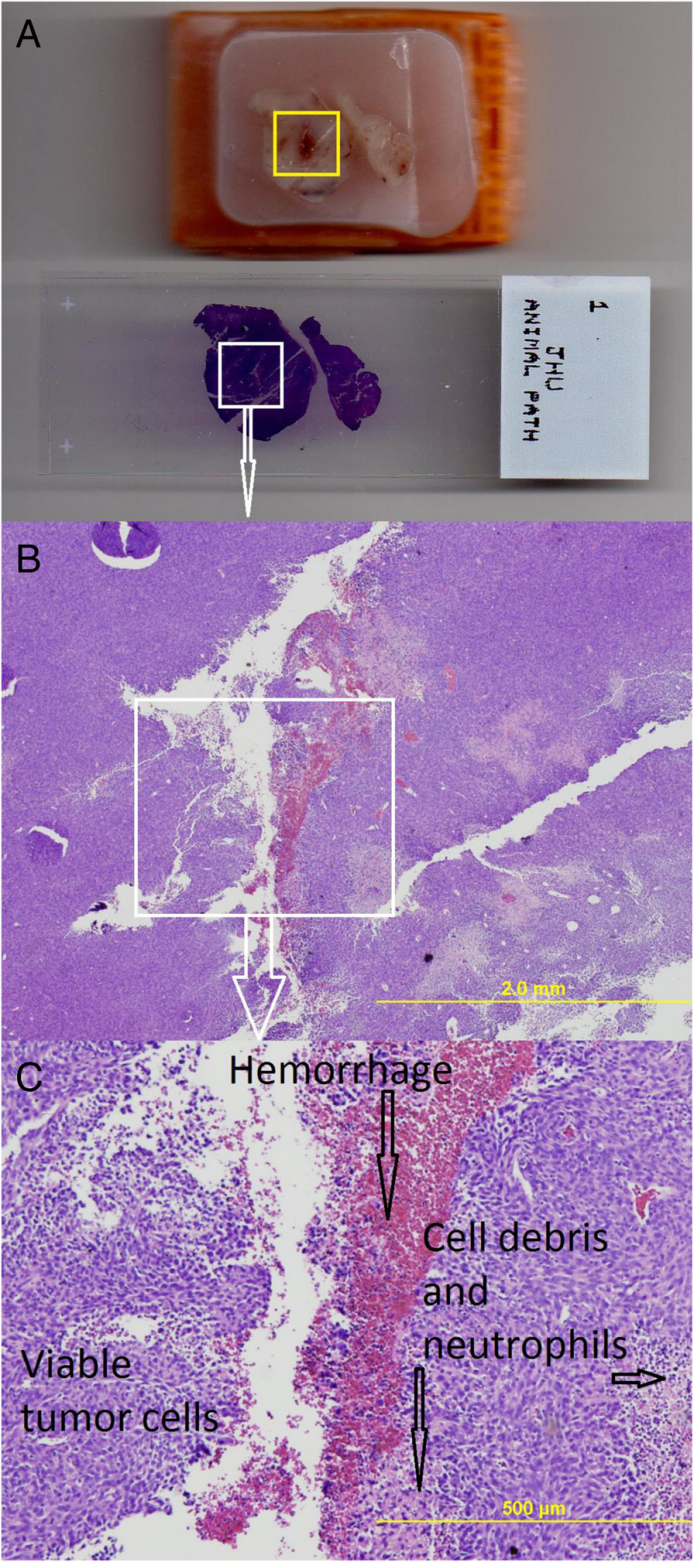
Pathological specimen. **a** A paraffin tumor tissue block with central necrosis (marked in *yellow square*) from reference rat 9 (*Top*) and its corresponding H&E stain (*bottom*). **b** The center of the H&E stain in ×20 magnification. There is a pathological correlation between the necrotic center on the H&E stain and the macroscopic morphology of the central necrosis (marked in *yellow square*) in the tissue block in Fig. 2 **a. c** The center of the H&E stain in ×80 magnification. The central necrotic area consists of hemorrhage, cell debris, and neutrophil infiltration

First, a pathologist reviewed the gross tumor tissue blocks in paraffin that was processed from rat 9—the reference rat, to identify and delineate the viable portion of the tumor by its macroscopic morphology, and the delineation was validated using microscopic examination of the associated H&E stains (Fig. 3). Next, the whole tumor area and its viable portion were delineated on each of the 51 macroscopic histological images, without review of the FDG-PET data. Thus, the examination of the unstained frozen sections for the fraction of viable tumor was informed by the detailed examination of the macroscopic and microscopic characteristics of the H&E-stained paraffin-embedded tissues from rat 9.

For each sectioning position, a corresponding cranial-caudal level in the FDG-PET images was determined using imaging bed position, and two FDG-PET slices closest to that level were selected. Then, the rostral FDG-PET slice was matched to the macroscopic histological image of the rostral cross-sectional surface and the caudal PET slice to the histological image of the caudal surface, thus forming two pairs of matched images with each pair containing a FDG-PET slice and its matched macroscopic histological image.

After each pair of matched images was established, the FDG-PET slice was registered to the histological image under the guidance of the fiducial markers using Amide software (http://amide.sourceforge.net/index.html). The macroscopic histological image acquired at 200 DPI had a pixel size of 0.127 × 0.127 mm^2^, which was much smaller than a FDG-PET slice’s axial pixel size of 1 × 1 mm^2^. In order for them to co-register, the smaller pixel size was adjusted to match the larger one, resulting in a coarser histological image with a lower resolution, as demonstrated in Fig. 2c, d.

### Analysis of image data

Four FDG-PET tumor contouring methods were investigated. The first three methods were based on fixed single value thresholds (voxel counts in our study), and the last method used an auto-detection algorithm (MIM software, Cleveland, Ohio), which was based on acceleration/deceleration of pixel intensity changes [15].

In method I, a spectrum of thresholds was determined by using 15–90 % (in 5 % increments) of the maximum single tumor voxel counts (C_max_) in each rat.

In method II, all 3 × 3 × 3 cubic voxel sets containing the C_max_ voxel were examined. Tumor peak counts (C_peak_) was defined as the highest average voxel counts among all 3 × 3 × 3 cubic voxel sets. A spectrum of thresholds was determined using 15–90 % (in 5 % increments) of C_peak_.

In method III, a liver mean voxel count (C_liver mean_) was defined as the average voxel counts in a sphere 6 mm in diameter, located in the right upper portion of the liver of each rat. The standard deviation (SD) of voxel counts in the sphere (C_liver SD_) was also calculated. A spectrum of thresholds was determined by a range of multiples of C_liver mean_ plus 2 × C_liver SD_.

In method IV, each rat’s FDG- PET image file was imported to the MIM workstation (Cleveland, OH), and the “PET/SPECT edge contour” function was used to create a 3D tumor contour. The operator had to manually seed the tumor center to start the algorithm.

In methods I–III, in each FDG-PET slice, a gross tumor area (GTA) was generated by all pixels equal to or above the threshold value. In method IV, a gross tumor volume (GTV) was automatically generated from the contiguous 3D set of edge points defined by MIM software.

In each pair of co-registered FDG-PET slice and its corresponding macroscopic histological image, different GTAs generated from methods I–III were tested against the viable area of the tumor in the corresponding histological image. Statistical calculations were performed using a binary classification system with the assistance of ImageJ (NIH) to assess each GTA’s performance on tumor delineation (http://rsb.info.nih.gov/ij/). The GTV contoured in method IV was analyzed separately.

### Statistical calculations

**Fig. 4:**
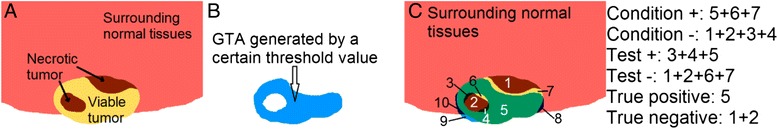
Binary classification system. A schematic diagram of our binary classification system: FDG-PET tumor contouring in a certain pair of co-registered images at a certain threshold value derived from a certain contouring method. **a** Macroscopic histological image representing a tumor with central and peripheral necrosis. The areas to be tested by FDG-PET are composed of both viable and non-viable portions of the tumor. Notably, the surrounding normal tissues are not included in the tested areas at this stage. **b** Gross tumor area (GTA) generated from a certain FDG-PET threshold value. **c** The fused image of **a** and **b**. Areas 8, 9, and 10 represent the overestimated areas in the GTA extending into the surrounding normal tissue and background air space

We designed a binary classification system to evaluate FDG-PET’s accuracy in contouring the viable and necrotic portions of the tumor on a macroscopic histological image. The test population—the areas to be tested—was the whole tumor areas (including both viable and necrotic potions of the tumor) delineated on the macroscopic histological image by the pathologist (WTA_H_), in units of pixels (Fig. 4a). The viable area of the tumor was considered as condition positive (validation standard) (Fig. 4c 5 + 6 + 7), and the necrotic area was considered as condition negative (Fig. 4c 1 + 2 + 3 + 4).

In each pair of co-registered images, different gross tumor areas (GTAs) were generated from the FDG-PET slice using methods I–III (Fig. 4b). Each GTA was tested on the histological image separately. The test outcome was positive (viable), if the histological area to be tested was included by the GTA (Fig. 4c 3 + 4 + 5), or negative (necrotic), if the area to be tested was not included by the GTA (Fig. 4c 1 + 2 + 6 + 7). As the test results for each histological area (in units of pixels) may or may not match that area’s actual viability status, true positive was defined as the viable areas of the tumor on the histological image that were correctly identified as viable by the GTA (Fig. 4c 5), and true negative was defined as the necrotic areas of the tumor that were correctly identified as necrotic (Fig. 4c 1 + 2). Sensitivity was defined as the viable areas correctly identified by GTA (true positive)/viable tumor areas delineated on histology (condition positive), and specificity was defined as the necrotic tumor areas correctly identified by GTA (true negative)/the necrotic tumor areas delineated on histology (condition negative). Notably, only the tumor areas on a histological image were tested in the design above. The surrounding normal tissues were excluded from the test population. To further evaluate the accuracy of each GTA, GTA_OE_ was defined as the overestimated areas in the GTA that extended beyond the whole tumor areas on the histological image (WTA_H)_ into the surrounding normal tissue and background air space (Fig. 4c 8 + 9 + 10).

**Fig. 5:**
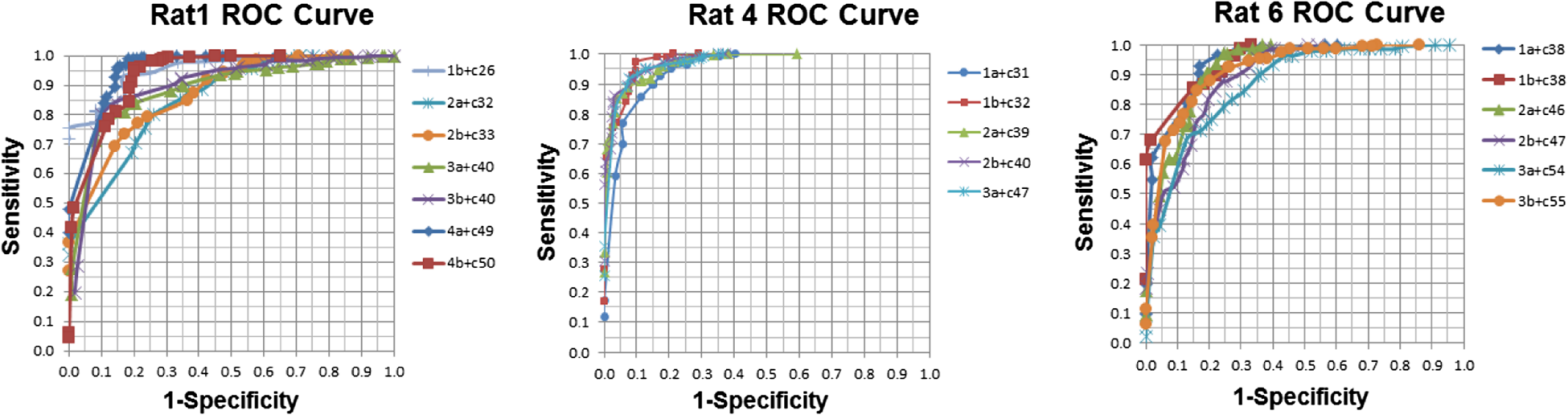
ROC curves. Eighteen ROC curves corresponding to 18 pairs of co-registered images from three representative rats are demonstrated here. The legend on the right of each illustration indicates the number of sectioning, the rostral/caudal surface (a/b), and the corresponding FDG-PET slice number

Different GTAs generated from methods I–III were applied to each of the 51 pairs of co-registered images, and the resulting sensitivity, specificity, and GTA_OE_ /WTA_H_ ratio (in percentage) were calculated. A receiver operating characteristic curve (ROC curve), which illustrated each contouring method’s performance on tumor delineation for each pair of co-registered images, was plotted using sensitivity and 1-specificity as *x* and *y* axes, respectively, with its area under the curve (AUC) approximated using the Gini coefficient (Fig. 5) [16]. Because in any particular pair of images, the GTAs generated from all three methods were all based on single value threshold (voxel counts) from the same FDG-PET slice, there was only one ROC curve for each image pair. In other words, in any particular pair of images, the ROC curves plotted from each of the three methods overlapped and complimented one another.

The generalized estimating equations (GEE) method was used to estimate the mean and standard error of the measurements, accounting for the correlations of the two cross-sectional surfaces from a single transverse sectioning [17]. Standard error of the mean (SE) and 95 % confidence intervals (CIs) were computed based on 10,000 bootstrap samples.

## Results and discussion

**Fig. 6:**
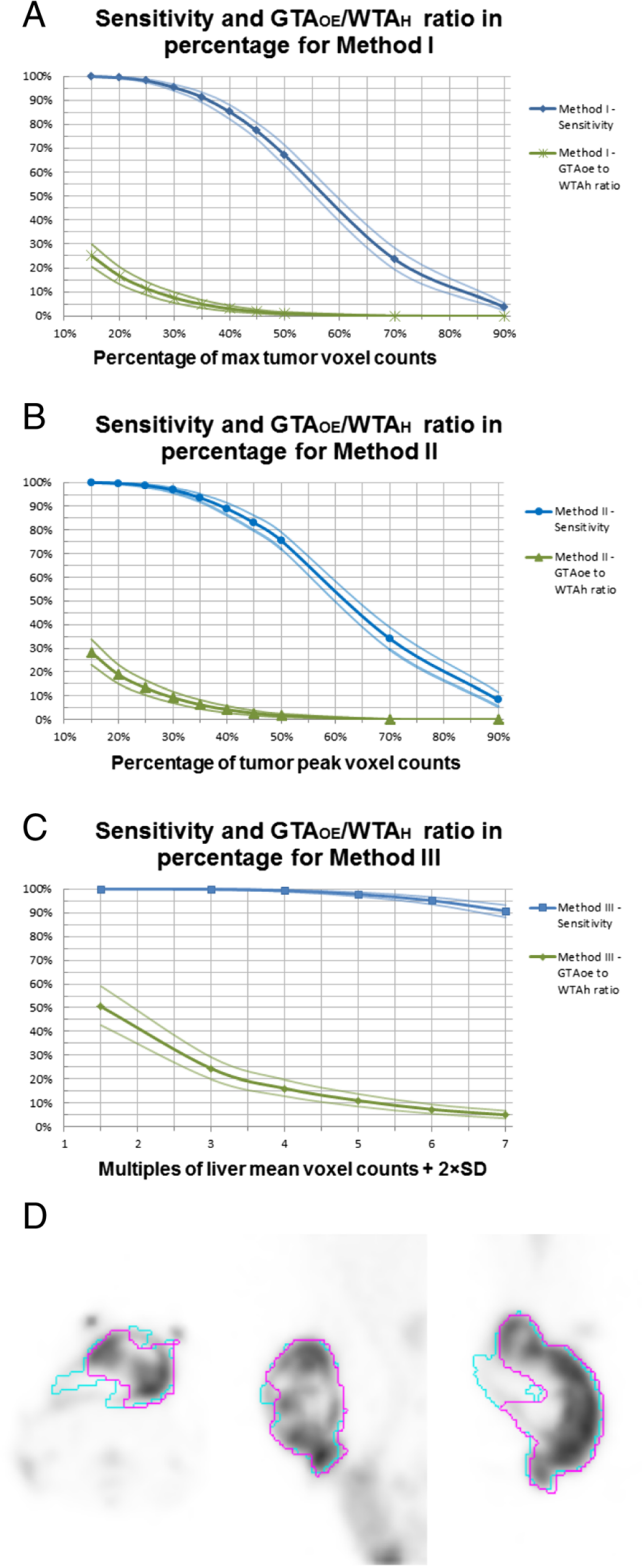
Sensitivity and GTA_OE_/WTA_H_ curves from methods I–III and MIM contouring in method IV. **a**–**c** The sensitivity and GTA_OE_/WTA_H_ were plotted against linear increment in threshold values derived from methods I–III, respectively. In each plot, the means were plotted in a darker line with data points, accompanied by the 95 % confidence intervals plotted in a thinner line without data points. **d** FDG-PET image of rat 5 was loaded into the MIM system as described in method IV. Two tumor contouring attempts (ROI *blue* and ROI *magenta*) using PET/SPECT edge contour function were demonstrated in axial (*right*), sagittal (*middle*), and coronal (*left*) planes. The two attempts were initiated with different seed points and yielded two inconsistent gross tumor volumes. *GTA*_*OE*_ the overestimated area in the gross tumor area, *WTA*_*H*_ whole tumor area on histology

In methods I–III, the means and 95 % CI of the sensitivity, specificity, and GTA_OE_ /WTA_H_ ratio from 51 pairs of images were calculated for each GTA generated from each contouring method, respectively (Tables 1, 2, and 3). The ROC curves of all pairs of images have a mean AUC of 0.934 (CI of 0.911–0.954). The means and 95 % CIs of the sensitivity and GTA_OE_/WTA_H_ ratio of GTAs generated from each of the three methods were plotted in Fig. 6a–c, respectively.

**Table 1:** Method I—percentage of maximal voxel activity

C_max_(%)	Sensitivity (CI)	Specificity (CI)	GTA_OE_/WTA_H_ (CI)
15	99.9 % (99.8–100.0 %)	36.3 % (25.9–46.8 %)	25.0 % (20.4–29.9 %)
20	99.4 % (99.1–99.7 %)	50.4 % (39.7–60.7 %)	16.7 % (13.2–20.5 %)
25	98.1 % (97.4–98.8 %)	61.1 % (51.6–70.2 %)	11.3 % (8.7–14.1 %)
30	95.3 % (93.9–96.6 %)	70.9 % (62.7–78.6 %)	7.5 % (5.5–9.8 %)
35	91.3 % (89.2–93.3 %)	77.5 % (70.3–84.2 %)	5.0 % (3.4–6.7 %)
40	85.3 % (82.3–88.2 %)	84.1 % (78.3–89.3 %)	3.1 % (2.0–4.4 %)
45	77.3 % (73.8–80.6 %)	90.0 % (85.8–93.8 %)	1.9 % (1.2–2.7 %)
50	67.2 % (62.9–71.4 %)	93.4 % (90.4–96.2 %)	1.1 % (0.6–1.7 %)
70	23.6 % (19.4–28.4 %)	99.4 % (98.9–99.8 %)	0.1 % (0.0–0.1 %)
90	3.7 % (2.3–5.4 %)	100.0 % (100.0–100.0 %)	0.0 % (0.0–0.0 %)

*CI* confidence interval, *GTA*_*OE*_ gross tumor area overestimate, *WTA*_*H*_ whole tumor areas on histology

**Table 2:** Method II—percentage of tumor peak activity

C_peak_ (%)	Sensitivity (CI)	Specificity (CI)	GTA_OE_/WTA_H_ (CI)
15	100.0 % (99.9–100.0 %)	32.2 % (23.0–42.2 %)	28.3 % (23.0–33.8 %)
20	99.6 % (99.4–99.8 %)	46.9 % (36.1–57.4 %)	18.9 % (15.0–23.0 %)
25	98.7 % (98.1–99.2 %)	57.4 % (47.2–67.1 %)	13.3 % (10.3–16.5 %)
30	96.9 % (95.9–97.8 %)	66.0 % (57.1–74.5 %)	9.3 % (7.0–11.8 %)
35	93.7 % (92.0–95.3 %)	73.8 % (65.8–81.1 %)	6.3 % (4.5–8.3 %)
40	89.0 % (86.4–91.5 %)	80.2 % (73.2–86.5 %)	4.2 % (2.8–5.8 %)
45	83.1 % (79.8–86.1 %)	86.3 % (80.9–91.2 %)	2.7 % (1.8–3.8 %)
50	75.6 % (72.0–79.1 %)	90.8 % (86.5–94.6 %)	1.7 % (1.1–2.5 %)
70	34.1 % (29.5–38.9 %)	98.9 % (98.0–99.6 %)	0.1 % (0.0–0.2 %)
90	8.4 % (5.5–11.6 %)	99.9 % (99.7–99.9 %)	0.0 % (0.0–0.0 %)

*CI* confidence interval, *GTA*_*OE*_ gross tumor area overestimate, *WTA*_*H*_ whole tumor areas on histology

**Table 3:** Method III—multiple of liver mean activity + 2 × standard deviation (SD))

Multiple of liver mean	Sensitivity (CI)	Specificity (CI)	GTA_OE_/WTA_H_ (CI)
1.5	100.0 % (100.0–100.0 %)	11.9 % (6.9–17.7 %)	50.5 % (42.7–59.1 %)
3	99.9 % (99.7–100.0 %)	36.9 % (26.8–47.1 %)	24.4 % (20.0–29.2 %)
4	99.3 % (98.8–99.6 %)	50.8 % (40.4–60.6 %)	16.2 % (13.0–19.8 %)
5	97.8 % (96.8–98.6 %)	62.1 % (53.0–70.7 %)	11.1 % (8.7–13.9 %)
6	95.1 % (93.5–96.5 %)	71.2 % (63.4–78.5 %)	7.4 % (5.6–9.5 %)
7	90.7 % (88.1–93.2 %)	78.1 % (71.5–84.3 %)	5.1 % (3.7–6.9 %)

*CI* confidence interval, *GTA*_*OE*_ gross tumor area overestimate, *WTA*_*H*_ whole tumor areas on histology

In method IV, the resulting GTV using MIM’s “PET edge” function was not reproducible. Depending on the position where the seed point was chosen and the direction it was dragged, the resulting tumor delineations were different (Fig. 6d).

Our study uses immunologically intact rats, which resemble human tumors in vivo. As animal irradiation systems become more common, more precise contouring methods of animal tumors are of experimental importance.

Our study demonstrates that single value threshold tumor contouring method using ^18^F-FDG-PET can reliably delineate the viable portion of a tumor, as illustrated by a mean AUC of 0.934 (CI of 0.911–0.954) (Fig. 5). There is some variation among the eight rats in the sensitivities and specificities calculated from the same threshold percentage using the same contouring method, as illustrated by the confidence intervals in Tables 1, 2, and 3. A possible explanation is the influence of tissue viability heterogeneity on the FDG-PET voxel counts. A single threshold value in the necrotic portion of a tumor may exclude the adjacent very thin rim of viable tumor areas, and very small tumor areas beyond the spatial resolution of micro-PET may not be contoured [18].

In our study, sensitivity represented how well a FDG-PET generated GTA included the viable tumor area delineated on histology. Fifty percent sensitivity indicated that 50 % of the viable tumor area was covered by the GTA. In radiotherapy planning, a high sensitivity is desired to irradiate most, if not all viable tumor volumes.

Specificity represented how well a GTA managed to stay within the viable tumor area, without extending into the adjacent necrotic tumor area. In radiotherapy planning, a more selective radiation field may reduce the dose to surrounding normal tissue. An interesting question to ask is the effect of irradiating the necrotic tumor tissue contoured by FDG-PET on whole tumor growth.

One common concern during radiotherapy is the possibility of irradiating the normal structures surrounding the tumor target. This issue is difficult to address by simple statistical analysis, because it is influenced by many factors including the size, shape, and location of the tumor target and the radiation sensitivities of the tumor and surrounding tissues. In our study, we used GTA_OE/_WTA_H_ ratio to illustrate the extent to which a particular GTA extended into the normal structures surrounding the primary tumor target.

The most desirable contouring method for radiotherapy planning would have a sensitivity (and to a lesser degree, a specificity) close to 100 %, with a GTA_OE_/WTA_H_ ratio close to 0 %. In our study, a common characteristic demonstrated in Fig. 6 was that as the threshold value of a particular contouring method decreases, its sensitivity is increased at the expense of an increased GTA_OE_/WTA_H_ ratio. That being said, certain threshold values achieved a sensitivity greater than 90 % with a GTA_OE_/WTA_H_ ratio less than 10 %: 30 % and 35 % of both maximal tumor voxel activity (C_max_) and tumor peak activity (C_peak_). These threshold values can be considered in creating gross tumor volume in radiotherapy planning requiring broad tumor coverage with relatively high priority of adjacent normal tissue preservation. Tumor contouring using 50 % of C_max_ or C_peak_ – commonly reported threshold values in the literature, had relatively low sensitivities of 67.2 %–75.6 % with very low GTA_OE_/WTA_H_ ratios of 1.1 %–1.7 %, and may be considered in sub-volume boosting, where a high dose of radiation is prescribed to the most metabolically active region of the tumor.

Successful radiotherapy decreases peak tumor activity, while the liver radioactivity is expected to remain relatively constant [19]. Tumor contouring methods based on liver activity, therefore, potentially could be particularly helpful in post-radiation re-planning situations. Tumor contouring based on PERCIST with 1.5 × C_liver mean_ + 2 × SD had a sensitivity of 100 % but a high GTA_OE_/WTA_H_ ratio of 50.5 % (CI 42.7–59.1) [20]. Tumor contouring using 6 × C_liver mean_ + 2 × SD had a sensitivity of 95.1 % (CI 93.5–96.5) and a GTA_OE_/WTA_H_ ratio of 7.4 % (CI 5.6–9.5 %), which was comparable to contouring using 30 and 35 % of C_max_ or C_peak_, and may be considered in radiotherapy planning where prior radiation was performed.

Although our study was limited to mammary tumor in an animal model, it provided a template approach to perform similar studies with other tumor types using different radiotracers. As more radiotracers become available, similar studies can be performed to facilitate their application in clinical settings. For example, such an approach could be used in ^18^F-fluoromisonidazole(FMISO)-PET to evaluate how FMISO characterizes tumor tissue hypoxia in vivo in animal model and provide a roadmap for further research on FMISO-based radiotherapy planning [21].

Several issues need to be discussed. The first issue regards the variation between macroscopic and microscopic pathologies. The frankly necrotic portion of a tumor is easy to differentiate macroscopically from the viable portion. However, the gray zone in between, where there is a mixture of tumor cells, immunological cells, and cell debris, is difficult to identify on macroscopic images. To be conservative, we included these areas in the viable portion of the tumor, although the clinical importance of these areas needs further research.

The second issue is related to the limitation of our imaging system. The spatial resolution of the animal micro-PET scanner is approximately 2 mm [12]. With a typical cell diameter at tens of microns, failure to detect small tumors is possible. This limits the accuracy of the data analysis, especially when the tumors are small. Our data analysis is also limited by partial volume effect, which is more prominent in small tumors. Utilizing reconstruction algorithm designed to improve image-space resolution, e.g., resolution modeling during reconstruction, could help to improve the quality of our PET imaging and decrease partial volume effect [22].

The third issue is that tumors vary substantially in their metabolic activities. Our tumor model has relatively high FDG uptake, and the variability in voxel counts may affect the optimal threshold for tumor contouring. In fact, FDG-PET tumor contouring may even be tumor specific, and human tumors may have lower FDG uptake.

Finally, the contouring methods investigated in our study are based on single value threshold. It is worth debating whether tumor volume is best delineated with single value. Our last method using an edge detection technique was an attempt to evaluate the performance of a non-single-value contouring method. However, due to the system’s lack of reproducibility, we were unable to reliably analyze the data. A potential future study is to investigate non-single-value contouring algorithms developed by other groups, compare their results to those obtained in this study, and explore other edge detection methods. In addition, CT-based tumor contouring, which is usually performed in clinical settings, was not investigated for comparison to PET-based contouring methods in our study. This is due to technical difficulty in acquiring an intravenously enhanced CT imaging of a rat while maintaining its anatomic orientation between two different imaging beds. Hybrid small animal PET/CT or PET/MR may provide better soft tissue characterization and additional useful information.

## Conclusions

Single-value threshold tumor contouring using ^18^F-FDG-PET is able to accurately delineate the viable portion of a tumor in this rodent model and provide an imaging blueprint to future animal radiotherapy planning studies seeking dose efficiency and reduction. Our study design may also provide a template approach to radiotracer validation in small animals and help to inform future clinical studies. While human tumors no doubt differ, our study suggests threshold values of 30 and 35 % of C_max_ or C_peak_ and 6 × Cliver + 2 × SD are all appropriate for delineating viable tumor volume in a rodent breast cancer model.

## Compliance with ethical standards

Ethical approval: All applicable international, national, and/or institutional guidelines for the care and use of animals were followed. This article does not contain any studies with human participants performed by any of the authors.
